# Commentary: Neural Control of Vascular Reactions: Impact of Emotion and Attention

**DOI:** 10.3389/fpsyg.2016.01613

**Published:** 2016-10-20

**Authors:** Rashmi Gupta

**Affiliations:** Swiss Center for Affective Sciences, University of GenevaGeneva, Switzerland

**Keywords:** baroreceptors, blood pressure, attention, emotion processing, load

Recent studies have claimed that processing of negative emotional information is modulated by the attentional demands of the task (Pessoa et al., 2002; Okon-Singer et al., 2007; Gupta and Srinivasan, 2015; Gupta et al., 2016). For example, Okon-Singer et al. (2007) manipulated low and high perceptual load of the primary letter-search task and used negative and neutral pictures as distractors. They found that negatively valenced distractors (compared to neutral) capture attention only in the low-load condition however, not in the high-load condition. These results fit with the perceptual load theory of attention for distractor processing (Lavie, 1995). According to Lavie, processing of distractors is only prevented if the perceptual load of the primary task is sufficiently high to exhaust available attentional capacity.

In parallel with behavioral studies, neuroimaging studies have shown that neural activity related to emotional stimuli is also modulated by availability of attentional resources (Pessoa et al., 2002; Okon-Singer et al., 2014). For example, recently Okon-Singer et al. (2014) investigated whether attention affects behavioral, neural, and vascular (blood pressure, BP) reactions to the processing of irrelevant emotional distractors in healthy individuals. They used negative and neutral pictures, from the International Affective Picture System (Lang et al., 2008; Lohani et al., 2013), as distractors in a letter search task. Distractor pictures were encircled by two or six letters producing low and high perceptual load condition, respectively. Participants were instructed to ignore the picture while discriminating a target (“X” or “N”) letter and pressing corresponding buttons as fast and accurately as possible. BP was continuously recorded simultaneously to fMRI acquisition and statistical analyses were conducted only on systolic BP.

They found that when attention was available for processing distractors (low-load), negative pictures (relative to neutral) interfered with letter search performance. Processing of these pictures activated several emotion processing brain areas such as visual cortex, anterior insula, amygdala, and orbitofrontal cortex and simultaneously decreased BP. In contrast, when less attention was available for processing distractors (high-load) these reactions were attenuated or diminished. Furthermore, high perceptual load (relative to low-load) boosted activation in attentional processing brain areas (frontoparietal regions). These results are parallel with the theories of selective attention emphasizing the role of attention in regulating behavioral and neural responses to processing of irrelevant emotional information (Pessoa et al., 2002). Furthermore, these findings extend the role of attention in regulating autonomous response related to the processing of negative emotions by showing altered BP reactions to emotional information.

Okon-Singer et al. (2014) discussed their results with reference to the BP response to emotional stimuli, the neural regions involved, and the influence of attention on these responses. However, the neurophysiological link between emotion and attention processing was not made explicit by Okon-Singer et al.'s (2014), study by which BP levels were reduced while processing negative distractors under the low-load condition. The present paper focused on a possible mechanism underlying this effect.

It has been suggested that high level of BP exerts a negative influence on overall negative emotional experience and reactivity (McCubbin et al., 2011). In support of this view, several studies have shown that higher BP is associated with dampened responses to negative emotional stimuli (Pury et al., 2004). This effect was also found to be valid for more general emotional personality traits (e.g., individuals with low and high levels of worry). For example, it has been found that low worry individuals displayed enhanced BP levels and high worry individuals displayed reduced BP levels (see Delgadoa et al., 2014 for a discussion).

Lower BP is believed to activate a facilitatory positive feedback pathway form the cardiovascular to central nervous system (CNS) mediated by the baroreceptors, enhancing stress, and emotional reactivity to negative information. This suggests that baroreceptor feedback pathway works when the level of BP is low. In Okon-Singer et al.'s (2014) study BP level got reduced because of the processing of negative information under low-load condition. In other words, processing negative information may cause transient stress/worry or negative mood induction in the low-load condition, which reduces the BP level. This could be the reason that why the baroreceptor feedback pathway work only under low perceptual load but not in the high-load. In contrast, higher BP activates an inhibitory negative feedback from the cardiovascular system to the CNS, reducing stress, and emotional reactivity to negative information. The Baroreceptors are sensors located in blood vessels, which regulate BP by detecting the amount of stretch of the blood vessel walls, and send the signal to the CNS for affective-emotional processing.

It has been suggested that the neuroanatomical bases for the CNS inhibitory effect of the baroreceptors comprises a central feedback pathway arising from the nucleus of the tractus solitarius (in the medulla oblongata) spreading to the reticular formation, hypothalamus, thalamus, central nucleus of the amygdala, ventral hippocampus, the periaqueductal gray, medial-lateral prefrontal, anterior insular, and anterior cingulated cortexes (Delgadoa et al., 2014; see Rau and Elbert, 2001; for a review). Through the connections to all these structures baroreceptor activation produces a generalized inhibitory effect on CNS functions. Notably, in Okon-Singer et al.'s (2014) study, a positive correlation was observed between neural activity in most of these brain areas and BP response for the processing of emotional stimuli in the low-load condition. Most of these regions (e.g., amygdala, prefrontal, and anterior cingulate cortex, insula), known to play a key role in emotion processing, were found to be involved in vascular reactions associated with stress (see Gianaros and Sheu, 2009 for a review). Thus, through this network, activation of the baroreceptors due to increased BP reduces reaction to negative emotional stimuli. In contrast, decreased BP enhances reaction to negative stimuli especially in low perceptual load condition found in Okon-Singer et al.'s (2014) study. This could be a relevant mechanism mediating the effect of BP in affective-emotional processing under the low-load condition observed in Okon-Singer et al.'s (2014) study (see Pury et al., 2004). This neurophysiological relation between emotion processing and attention was not made explicit by Okon-Singer et al.'s (2014), despite the significant implications it has for the role of attention in emotion processing. Since blood pressure is the product of cardiac output and systemic vascular resistance (and cardiac output is the product of stroke volume and heart rate), the vascular contribution to these processes should be determined by further studies controlling for other hemo/cardiodynamic variables.

## Author contributions

RG has written the manuscript.

### Conflict of interest statement

The author declares that the research was conducted in the absence of any commercial or financial relationships that could be construed as a potential conflict of interest.
